# 1What do first-time mothers worry about? A study of usage patterns and content of calls made to a postpartum support telephone hotline

**DOI:** 10.1186/1471-2458-10-611

**Published:** 2010-10-15

**Authors:** Hibah Osman, Monique Chaaya, Lama El Zein, Georges Naassan, Livia Wick

**Affiliations:** 1 Department of Health Behavior and Education, American University of Beirut, Beirut, Lebanon; 2 Department of Epidemiology and Population Health, American University of Beirut, Beirut, Lebanon; 3 Department of Family Medicine, Columbia University, New York, New York, USA; 4 Neurologic Institute, University Hospitals Case Medical Center, Cleveland, Ohio, USA; 5 Department of Social and Behavioral Sciences, American University of Beirut, Beirut, Lebanon

## Abstract

**Background:**

Telephone hotlines designed to address common concerns in the early postpartum could be a useful resource for parents. Our aim was to test the feasibility of using a telephone as an intervention in a randomized controlled trial. We also aimed to test to use of algorithms to address parental concerns through a telephone hotline.

**Methods:**

Healthy first-time mothers were recruited from postpartum wards of hospitals throughout Lebanon. Participants were given the number of a 24-hour telephone hotline that they could access for the first four months after delivery. Calls were answered by a midwife using algorithms developed by the study team whenever possible. Callers with medical complaints were referred to their physicians. Call patterns and content were recorded and analyzed.

**Results:**

Eighty-four of the 353 women enrolled (24%) used the hotline. Sixty percent of the women who used the service called more than once, and all callers reported they were satisfied with the service. The midwife received an average of three calls per day and most calls occurred during the first four weeks postpartum. Our algorithms were used to answer questions in 62.8% of calls and 18.6% of calls required referral to a physician. Of the questions related to mothers, 66% were about breastfeeding. Sixty percent of questions related to the infant were about routine care and 23% were about excessive crying.

**Conclusions:**

Utilization of a telephone hotline service for postpartum support is highest in the first four weeks postpartum. Most questions are related to breastfeeding, routine newborn care, and management of a fussy infant. It is feasible to test a telephone hotline as an intervention in a randomized controlled trial. Algorithms can be developed to provide standardized answers to the most common questions.

## Background

The postpartum period is stressful for many mothers. Concerns about health and parenting abilities are common [1,2]. The lack of predictability of the infant, sleep disturbances, body changes, and lifestyle restrictions have also been documented [3]. First-time mothers have the added stressor of adaptation to their new role as mothers. These issues may have a significant impact on maternal confidence, self-esteem and overall emotional wellbeing.

Telephone hotlines have been widely used to provide immediate support for a variety of medical, psychological and social conditions. The accessibility and anonymity that the telephone provides makes it a useful tool for organizations seeking to provide information, counseling, and support to a target population. In the field of Maternal Child Health, hotlines have been utilized to support mothers during pregnancy and after delivery [4]. Many hotlines were developed in response to early discharge policies to provide continuity and support to parents after discharge from the hospital [5,6]. Some hotlines were established exclusively as resources for lactation support [7,8]. Others targeted mothers considered to be at risk for complications [9]. Few studies have examined the utilization of hotlines by new mothers and the types of questions asked by callers.

Lebanon is a middle-income country in the Middle East with a population of 4 million and a fertility rate of 2.2% [10]. The use of hotlines in Lebanon has been limited. Hotlines have been established for sexual health, domestic violence and breastfeeding. An informal survey of these hotline services revealed that the volume of calls varies between a 10 to 15 calls per day to a call every two weeks depending on recent marketing efforts.

About 90% of all deliveries in Lebanon occur in hospitals [11]. Women general remain hospitalized for 24 - 48 hours after vaginal delivery and for 48 - 72 hours after cesarean section. The postpartum visit is usually scheduled at 40 days postpartum. There are no local guidelines to related to the content of the postpartum visit and there is great variability among physicians in terms of the issues that are addressed during that visit. Infants are usually seen at 1 - 2 months of life for the first well baby visit. The visit usually includes vaccinations, a physical exam with focus on growth and development, as well as anticipatory guidance for the parents. There are currently no community services that provide social or medical support to new mothers or their infants outside of the routine medical visits. Women seeking answers to their questions related to self-care or newborn care in the postpartum period usually rely on their social network or call their physicians for answers. Mothers who depend on their social network can be confused by the conflicting or inaccurate information they receive. When women depend on their physicians for answers, the increased workload of responding to telephone calls can be a burden.

We were interested in establishing a hotline for postpartum support for first-time mothers in an attempt to reduce the stress associated with that phase of life. However, the low utilization of existing hotlines and concerns about whether women would trust the information provided by such a service lead us to question whether women would utilize the hotline as a source of information. This study was conducted in preparation for a larger trial designed to evaluate the impact of different interventions on reducing stress in the postpartum for healthy first-time mothers with healthy infants. The purpose of this study was to examine the utilization of a hotline dedicated to postpartum support by first-time mothers in Lebanon. Our aim was to clarify usage patterns and the types of questions asked as well as the perceived value of such a service.

## Methods

The study targeted first-time mothers delivering in hospitals throughout Lebanon. Hospitals with the highest volume of deliveries in each of the five Lebanese governorates were approached for participation in the study. Of the 23 hospitals approached 17 agreed to participate. Administrators in the hospitals that refused to participate cited concerns about patient privacy. The study protocol was approved by the Institutional Research Board at the American University of Beirut.

Recruitment began in early November 2007 and continued for two months until 350 women were enrolled in the study. All healthy first-time mothers who delivered healthy infants in participating hospitals during the study period were invited to participate. Women with chronic health conditions or prenatal, delivery or postpartum complications were excluded from the study. Mothers who had a preterm delivery or whose infant required admission to the Neonatal Intensive Care Unit were also excluded.

Women were recruited after delivery and prior to discharge. Recruiters were midwives, nurses or social workers. All received training in the consent process and completion of data collection tools. They were required to visit each hospital on a daily basis. At each visit, recruiters collected information on all new deliveries including parity, age of mother, date of delivery, and type of delivery from the delivery log. All eligible first-time mothers were approached for participation in the study.

Verbal consent was considered adequate since no identifying information was collected about participants and anonymity was ensured. Women who agreed to participate in this study were given a card on which the hotline number, study identification (ID) number and instructions on use of the hotline were recorded. The study identification number was linked to the delivery information of study participants.

The hotline was a mobile telephone number that women could call at anytime of the day or night with questions regarding parenting, infant care or self care during the first four months after delivery. The service was free of charge. Mothers were asked to send a text message or call the hotline and hang up after the first ring and a certified midwife would return their call within ten minutes. Mothers using the hotline could identify themselves by using their unique ID number. Study participants were neither encouraged nor discouraged from sharing the hotline number with other women. If women who had not been recruited into the study called the hotline, the baseline demographic and delivery information was collected at the time for the first call and they were given a new study ID number to utilize during future calls.

A full-time midwife was recruited to answer the hotline. She had experience in midwifery and lactation support and is a mother to two children. Algorithms were developed to address questions that we anticipated the midwife would have to answer. The algorithms were designed to allow the midwife to answer questions in a consistent manner based on the best available scientific evidence. In a setting where different people may answer the hotline, algorithms can provide consistency in responses and ensure that all questions are answered based on best evidence. Our algorithms were developed using a combination of medical journals, medical textbooks and expert opinion. A total of 17 algorithms related to baby care and 12 algorithms related to the mother were developed. These included topics such as infant feeding and elimination, baby blues and postpartum depression, contraception and resumption of menstruation. The midwife was given the algorithms in a folder and trained to use them. She was also trained on data collection and handling of telephone calls using role-play techniques. All medical complaints were referred to the patient's physician, and no diagnosis or treatment were given through the hotline. A family physician was available to support and back up the midwife for the duration of the study.

A semi-structured form was used to document data regarding the calls. Information collected included: ID number of the caller, date of delivery, date, time and duration of the call, questions asked, answers given, whether an algorithm was used to answer the question, and whether the caller was referred to her physician. At the end of each call, all first-time callers were asked whether they were satisfied with the service and whether they would use the service again.

Most callers asked more than one question per call. Data were analyzed using the caller, the call, or questions as the unit of analysis. Questions asked were categorized according to topic. Descriptive statistics (mean, median, mode and range or frequency distributions) on all the variables were calculated. Comparison of characteristics of callers and non-callers was performed using the chi-squared test and T-Test for qualitative and quantitative data respectively. Significance level was set at 5%. Data entry and analysis were conducted using SPSS version 16.

## Results

### The Callers

A total of 376 women were approached and 353 (94%) consented to participate in the study. Table 1 shows the demographic characteristics of all study participants according to whether they utilized the hotline or not. Twenty four percent (84/353) of those enrolled used the hotline. The mean age of the women who enrolled in the study was 25 ± 5 years and 74.4% reported that they intended to breastfeed exclusively at the time of enrollment. Callers had a significantly larger mean number of prenatal visits than non-callers. Otherwise, no differences were noted between callers who utilized the hotline and those who did not.

**Table 1:** Characteristics of women enrolled in the study

	Total recruited (353) N (%)	Callers (84) N (%)	Non-callers (269) N (%)	P value
**Region of delivery**				
Greater Beirut	125(35.4)	34(40.5)	91(33.8)	
South	55(15.6)	15(17.9)	40 (14.9)	0.198
Bekaa	70(19.8)	10 (11.9)	60(22.3)	
North	103(29.2)	25 (29.8)	78(29.0)	
Education*				
None + Elementary	32(9.1)	5(6.0)	27(10.0)	0.107
Intermediate+ secondary	213(60.3)	46(54.8)	167(62.1)	
University degree	108(30.6)	33 (39.3)	70(27.9)	
**Age (yrs)**				0.123
Less than 21	70(19.8)	13 (15.5)	57(21.2)	
21-24	107(30.3)	20(23.8)	87(32.3)	
25-28	94(26.6)	31(36.9)	63(23.4)	
29-32	41(11.6)	11(13.1)	30(11.2)	
More than 32	41(11.6)	9(10.7)	32(11.9)	
**Type of delivery**				
Vaginal	191(54.4)	42(50.6)	149 (55.6)	0.425
C- section	160(45.6)	41(49.4)	119(44.4)	
**Intent to breastfeed exclusively**	261(74.4)	68(81.0)	193(72.3)	0.113
Yes	90(25.6)	16(19.0)	74(27.7)	
No				
**Number of prenatal visits**				
Mean (± SD)	10 ± 4	10.85 ± 4.2	9.27 ± 3.7	0.001

• Elementary education refers to first though fifth grade;

• Intermediate education refers to sixth through eighth grade;

• Secondary education refers to eleventh though thirteen grade.

All callers reported that they were satisfied with the service and would encourage other women to use it. Approximately 60% (50/84) of women called at least once after their initial call. Twelve women who were not recruited into the study called the hotline after obtaining the number from a friend.

### The Calls

The total number of the calls received was 312. The mean number of calls per caller was 2.96 calls (Mode = 1; Median = 2). This excludes two outliers who called 24 and 43 times each. Most calls (89.1%) were made by the mother herself, 7.7% were made by her husband, 2.9% were made by her mother. The mean number of questions asked in each call was 2 ± 1. The algorithms were used to answer at least one of the questions asked in 62.8% of the calls. The midwife made follow up calls in 7.7% of the total calls and referred the caller to a physician in 18.6% of the calls.

The midwife received an average of three calls per day. The mean duration of the calls was 6 min ± 5 minutes (Mode = 3 min; Median = 4 min 40 sec; Range = 0.5 min - 39 minutes). The mean time of calls was at 13:10 hours. Four percent of the calls were received between 11 pm and 6 am. The mean peak time at which mothers called the hotline was 18 days postpartum (Mode = 3; Median = 8; Range = 0 - 116 days).

### The Questions

A total of 570 questions were asked during the 312 calls of which 139 were mother related and 377 were infant related. Table 2 shows the frequency distribution of different questions. Of the questions related to mothers 66% were about breastfeeding. Sixty percent of questions related to the infant were related to routine care such as normal patterns of feeding and elimination, sleep patterns, umbilical care, circumcision care or pacifier use. Questions related to fussiness and crying comprised 23% of the infant related questions.

**Table 2:** Distribution of questions by topic (n = 570 questions)

Mother related questions (n = 193)	N (%)	Infant related questions (n = 377)	N (%)
Breastfeeding technique	81 (42)	Normal infant care	227 (60)
Breastfeeding problem	46 (24)	Feeding/Elimination/regurgitation (n = 106)	
Medical Problem	17 (9)	Sleep issues (n = 25)	
Other issues	14 (7)	Circumcision/cord care (n = 14)	
Body image	11 (6)	Pacifier use (n = 2)	
Wound care/hygiene	10 (5)	Others questions related to normal care (n = 80)	
Fatigue	5 (3)	Fussy infant	86 (23)
Return to work	3 (2)	Medical problem	24 (6)
Family pressure	3 (2)	Skin problem/diaper rash	21 (6)
Tearfulness/low mood	3 (2)	Other issues	19 (5)

**Figure 1:**
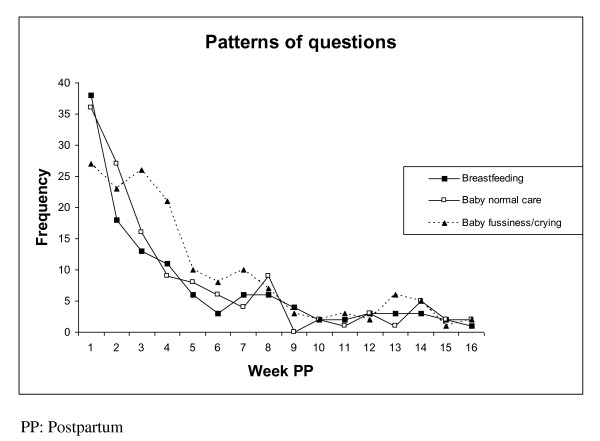
**Frequency of questions asked by timing postpartum**.

Figure 1 shows the types of questions asked according the timing postpartum. Questions related to breastfeeding were highest immediately after delivery and decreased gradually until week seven postpartum. Calls related to fussiness of the infant were highest in the first three weeks then decreased slowly until the ninth week. Questions about infant care were asked most frequently in the first three to four weeks postpartum.

## Discussion

The characteristics of the study sample as described in Table 1 are reflective of the population of women delivering in Lebanon. Recent studies on maternity and birth in Lebanon have consistently shown that women have a high educational level and intent to breast feed exclusively is high. Studies by other researchers as well as insurance companies consistently yield a cesarean section rate of 45 - 49%.

In our population, 24% of first-time mothers who were given access to a hotline service for postpartum support utilized the service. This utilization rate is similar to that obtained by Rush in a study in Ontario, Canada [5]. Higher utilization by women who had more prenatal visits could mean that women who access health services more often are more likely to access hotlines when they are available. This utilization rate is despite the fact that women did not know the person responding to their calls. Hotlines established in the hospitals and targeting their own patients could see a higher utilization rate.

Our users reported that they were satisfied with the service. This is supported by our finding that over half of the callers called the hotline repeatedly and that women shared the hotline number with friends or relatives who were not enrolled in the study.

We found that the volume of calls was highest in the first four weeks after delivery and decreased dramatically after the eighth week postpartum. The fact that most calls were before the time for a postpartum visit or well baby visit supports the value of giving women access a hotline for information. Many of the questions asked were for reassurance or validation that what the parent is doing is right. Having access to immediate answers in these situations could potentially have a significant impact on stress and confidence levels. Our algorithms covered a good proportion of the questions asked and few had to be referred to a physician.

Based on our experience, the volume and timing of calls by a limited number of study participants was not overwhelming and could potentially be handled by someone with other responsibilities working on a nursing floor. Budgeting and cost calculations should include a telephone line and one nurse or midwife. However, if a hotline is planned to cover a larger population of women, management of the service would need to be planned based on expected call volumes. Seigel and colleagues note from their study on a maternity in California that with time the number of calls grew to exceed three times the number of deliveries [6]. If the volume of calls is high, resources required for management of such a hotline would need to be reassessed.

Like other studies, we found mothers seek information about normal infant care and reassurance that their experience in "normal" [5,6]. Struggles with breastfeeding and causes of fussiness in the newborn also emerged as common sources concern for mothers. Although we cannot estimate the impact that our hotline had on breastfeeding success, the volume of questions related to breastfeeding support the need for such service.

## Conclusions

This is a descriptive study of hotline use and we have demonstrated that when offered this service, women use it and appreciate the service. To justify the allocation of resources to provide such a service, governments or hospitals would need data to support that this hotline improves outcomes such as breastfeeding success or the health and wellbeing of parents or their infants. Our experience demonstrates that hospitals can provide this service to their patients without adding a significant workload to nurses. Nurses on duty can answer questions, and algorithms can be used to standardize answers. We found the utilization rate of the hotline high enough to justify including it as an intervention on our randomized trial.

## Competing interests

The authors declare that they have no competing interests.

## Authors' contributions

The study was conceived by all authors. HO, LEZ and MC conducted the data analysis. HO, LEZ, GN and MC contributed to writing of the manuscript. All authors read and approved the final manuscript.

## Pre-publication history

The pre-publication history for this paper can be accessed here:

http://www.biomedcentral.com/1471-2458/10/611/prepub
